# Permeability of Ibuprofen in the Form of Free Acid and Salts of L-Valine Alkyl Esters from a Hydrogel Formulation through Strat-M™ Membrane and Human Skin

**DOI:** 10.3390/ma14216678

**Published:** 2021-11-05

**Authors:** Joanna Klebeko, Paula Ossowicz-Rupniewska, Anna Nowak, Ewa Janus, Wiktoria Duchnik, Urszula Adamiak-Giera, Łukasz Kucharski, Piotr Prowans, Jan Petriczko, Norbert Czapla, Piotr Bargiel, Marta Markowska, Adam Klimowicz

**Affiliations:** 1 Department of Chemical Organic Technology and Polymeric Materials, Faculty of Chemical Technology and Engineering, West Pomeranian University of Technology in Szczecin, Piastów Ave. 42, 71-065 Szczecin, Poland; joanna.klebeko@gmail.com (J.K.); ejanus@zut.edu.pl (E.J.); 2 Department of Cosmetic and Pharmaceutical Chemistry, Pomeranian Medical University in Szczecin, Powstańców Wielkopolskich Ave. 72, 70-111 Szczecin, Poland; anowak@pum.edu.pl (A.N.); wiktoria.duchnik@pum.edu.pl (W.D.); lukasz.kucharski@pum.edu.pl (Ł.K.); adklim@pum.edu.pl (A.K.); 3 Department of Pharmacokinetics and Therapeutic Drug Monitoring, Pomeranian Medical University in Szczecin, 70-111 Szczecin, Poland; uadamiak-giera@pum.edu.pl; 4 Department of Plastic, Endocrine and General Surgery, Pomeranian Medical University in Szczecin, Siedlecka 2, 72-010 Police, Poland; piotr.prowans@pum.edu.pl (P.P.); jan.petriczko@pum.edu.pl (J.P.); norbert.czapla@pum.edu.pl (N.C.); piotr.bargiel@pum.edu.pl (P.B.); markowskamh@gmail.com (M.M.)

**Keywords:** derivatives of ibuprofen, Franz cell diffusion, transdermal delivery system, Celugel^®^, human skin, Strat-M™

## Abstract

This paper aimed to evaluate the effect of vehicle and chemical modifications of the structure of active compounds on the skin permeation and accumulation of ibuprofen [IBU]. In vitro permeation experiments were performed using human abdominal skin and Strat-M™ membrane. The HPLC method was used for quantitative determinations. The formulations tested were hydrogels containing IBU and its derivatives and commercial gel with ibuprofen. The results obtained indicate that Celugel^®^ had an enhancing effect on the skin penetration of IBU. The average cumulative mass of [IBU] after 24 h permeation test from Celugel^®^ formulation through human skin was over 3 times higher than for the commercial product. Three ibuprofen derivatives containing [ValOiPr][IBU], [ValOPr][IBU], and [ValOBu][IBU] cation were evaluated as chemical penetration enhancers. The cumulative mass after 24 h of penetration was 790.526 ± 41.426, 682.201 ± 29.910, and 684.538 ± 5.599 μg IBU cm^−2^, respectively, compared to the formulation containing unmodified IBU-429.672 ± 60.151 μg IBU cm^−2^. This study demonstrates the perspective of the transdermal hydrogel vehicle in conjunction with the modification of the drug as a potential faster drug delivery system.

## 1. Introduction

Topical medications, including non-steroidal anti-inflammatory drugs, are widely accepted. This is due to their relatively good efficacy with a better safety profile compared to its oral counterparts. However, the high efficacy of different topical preparations is related to a combination of both pharmacokinetics and pharmacodynamics properties of the various drugs and their formulations. Therefore, more and more often, new methods are sought to increase drug penetration through the modification of the active compound or appropriate choice of the vehicle.

Skin permeation study is an important element in modeling vehicles forms containing drugs [1]. However, the penetration degree of active substances may be different depending on the compounds’ physical and chemical properties as well as the vehicle used [2]. In recent years, the transdermal drug supply has been gaining more and more interest. Such use of drugs above all provide controlled and prolonged substance active release into the systemic circulation. Moreover, advantages associated with transdermal delivery also include non-invasive delivery, reduced dosing frequency, constant levels of the drug in the plasma, and reduced drug toxicity and adverse events [3]. The main barrier limiting the penetration of active substances through the skin is the stratum corneum (SC), which prevents excessive water loss, and the entry of microorganisms, allergens, and chemical substances [2,4,5]. The SC is a thin membrane consisting primarily of cornified epidermal [6] cells, while the main components are lipids, such as ceramides, fatty acids, and cholesterol [7]. As is well known, the skin is a natural protective barrier. The outer layer of the skin—SC—protects against the penetration of hydrophilic substances, while the viable epidermis, situated below the SC, prevents the penetration of more lipophilic compounds [8]. In order to increase the penetration of drugs through the SC, the active substances are often modified, which, in combination with a well-chosen vehicle, can in bigger amounts penetrate through the hardly permeable human skin [6].

Ibuprofen [IBU] [(*RS*)-2-(4-(2-methylpropyl)phenyl)propanoic acid] is a small molecule (MW = 206 g·mol^−1^) with an octanol-water partition coefficient (log P) of around four [8], and it is a popular non-steroidal anti-inflammatory drug (NSAID); it is commonly used to treat osteoarthritis and rheumatoid arthritis, pain, and fever [2,5,9]. The oral delivery route has been the most widely used; however, this drug is very often applied topically to the skin. In view of the poor skin permeability of ibuprofen, there are various ways to ensure effective dosage of the drug at application. This is to be ensured by, among others, appropriate drug vehicles. In addition, the topical use of this drug is one of the most important methods of delivering it to the body and is an attractive alternative to conventional methods such as oral routes. In the topical application of NSAIDs, the active substance must penetrate the skin rapidly into the underlying layers in order to achieve a rapid therapeutic effect [6]. In the literature, one can find more and more reports of an additional modification of NSAIDs with respect to increasing their percutaneous transport [2,10,11]. This may also be the case for ibuprofen, which is characterized by low solubility (21 mg·dm^−3^ at 25 °C in water) as well as relative high lipophilicity, which results in its low permeation through the skin [4]. In our previous studies, we have shown that ion pairs of L-valine alkyl esters, with the length of chain alkyl from methyl to octyl, show higher penetration through pigskin from alcohol vehicles (methanol, ethanol, and isopropanol) [6]. As it is known, the applied vehicle has a great influence on the penetration of the active substance through the skin. Other dosage forms of ibuprofen and other non-steroidal anti-inflammatory drugs are also known, such as electrospun nanofibers [12,13,14,15].

Ibuprofen is the active ingredient in many topical formulations. This route of administration is used to reduce unwanted side effects and avoid first-pass metabolism in the liver and minimize gastrointestinal side effects [16]. However, due to its poor penetration capacity through the stratum corneum, it is difficult to obtain an effective concentration [17].

Hydrogels are of great interest to scientists in the field of cosmetology and dermatology. The main advantages of their use include the ease of application and significant minimization of side effects [18]. In recent years, more and more transdermal hydrogels with a composition that favors faster transport of drugs have also appeared. One of such preparations is the Celugel^®^ (Actifarm, Poland), which is a hydrophilic gel base containing water, glycerol, and a gelling agent: hydroxyethyl cellulose (HEC). The Celugel^®^ is intended for the preparation of drugs applied both to the skin and mucous membranes [19].

In our present study, we examined the effect of the structural modification introduced into the ibuprofen molecule on the permeability from the gel vehicle—Celugel^®^—through the human abbreviation and Strat-M™ membrane.

## 2. Materials and Methods

### 2.1. Chemicals

The following components were used in this research: acetic acid, potassium chloride, propan-2-ol, methanol, and sodium chloride (analytical grade, Chempur, Piekary Śląskie, Poland); acetonitrile (HPLC grade, J.T. Baker, Phillipsburg, NJ, USA); ethanol (p.a., Linegal Chemicals, Warszawa, Poland); disodium phosphate (p.a.); potassium dihydrogen phosphate (p.a.); Strat-M™ membrane (Merck, Darmstadt, Germany); Celugel^®^ (Actifarm, Warszawa, Poland); and commercial product (in gel form) (Dolorgiet Pharmaceuticals, Bonn, Germany).

### 2.2. Ibuprofen and Its Derivatives

Ibuprofen (as reference substances) and L-valine alkyl esters, obtained on their own, were used in this research. Esters with chain lengths from C1 to C8 (including isopropyl chain) were used in this research. The synthesis of ibuprofenates of amino acid alkyl esters was conducted as described previously [2,6].

### 2.3. Production of Hydrogel with Active Pharmaceutical Ingredient

**Figure 1:**
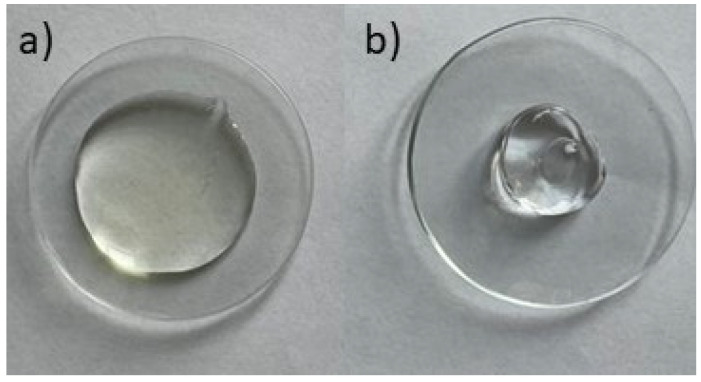
The appearance (**a**) of the vehicle used for this research and (**b**) a commercial product.

To prepare hydrogel, ibuprofen, or its derivatives, they were accurately weighed and transferred to porcelain mortar and levigate with an appropriate amount of ethanol 96% (v/v) to form a smooth paste. The appropriate amount of Celugel^®^ was then added to each trial and mixed for 2 min. The finished gel was transferred to the package and stored at room temperature. The studies were carried out in triplicate for each compound. Compositions of the hydrogels developed are provided in Table 1. Figure 1 shows the appearance of the pharmaceutical substrate used in the research—Celugel^®^ (a) and a commercially available hydrogel containing IBU (b). The concentration of active ingredient (expressed as ibuprofen content) was the same for all prepared formulations and used commercial product and was 5%.

### 2.4. In Vitro Permeation Studies

The in vitro permeation experiments were carried out using Franz-type diffusion cells (SES GmbH Analyse Systeme, Bechenheim, Germany) across human skin and Strat-M™. Human skin was excised from the abdomen of living patients as a result of plastic surgery. The study was approved by the Ethical Committee of Pomeranian Medical University in Szczecin (KB0012/02/18). The skin of 0.5 mm in thickness was divided into about 2 cm × 2 cm pieces. When not used immediately, the skin was wrapped in aluminum foil and kept refrigerated (−20 °C) for up to 3 months, which is considered a safe amount of time to maintain the skin’s barrier properties [20]. Before using for permeation study, the skin samples were slowly thawed at room temperature and were hydrated by PBS pH 7.4 [6,8,9] followed by impedance measurement (see the section below). The available diffusion area was 1 cm^2^, and the volume of the acceptor chamber, containing PBS solution (pH 7.4) thermostatted at 32.0 ± 0.5 °C, was about 8 cm^3^. Each time, 1 g of a hydrogel containing 5% of the tested compound was placed in the donor chamber. The amount of 0.5 cm^3^ of samples was taken from the acceptor chamber at specified time intervals (1, 2, 3, 5, 8, and 24 h) and immediately refilled with the same volume of fresh solution.

### 2.5. In Vitro Skin Accumulation

After the permeation study was completed, the skin was removed from the Franz cell, then washed with a 0.5% solution of sodium lauryl sulfate, and dried with a paper towel. After air drying, the skin was cut into small pieces and extracted with 2 mL of methanol for 24 h at 4 °C. Then, the samples were homogenized for 3 min using a homogenizer (IKA^®^T18 digital ULTRA TURRAX, Staufen, Germany) and centrifuged at 3500 rpm for 5 min. After centrifugation, the solution was analyzed by the HPLC method. The accumulation of the IBU and its salt in human skin and Strat-M™ membranes was calculated by dividing the amount of the substances remaining in the samples by mass of skin or Start-M membranes sample and was expressed as the mass of IBU mass of the skin (µg·g^−1^).

### 2.6. HPLC Analysis

The [IBU] and its salt concentrations in the acceptor phase were measured by the HPLC method. For this purpose, a Knauer liquid chromatograph (Berlin, German) was used, equipped with the WellChrom model K1001 pump, model K2600 UV detector, a Rheodyne model 7125 injector, and Hypersil ODS (C18) column (particle size 5 µm; 125 × 4.0 mm I.D.; Thermo Scientific™ Waltham, MA, USA). For instrument control and data processing, EuroChrom 2000 for Windows software was applied. The column temperature was set at 25 °C. The mobile phase consisted of 0.02 mol·dm^−3^ potassium dihydrogen phosphate-acetonitrile-methanol (45/45/10, v/v/v), and the flow rate was 1 cm^3^·min^−1^. Chromatograms were collected at a wavelength of 264 nm. Each sample (injection size of 20 µL) was analyzed in triplicate, and the results are presented as mean value ± standard deviation (SD).

### 2.7. Skin Impedance

Skin electrical impedance was performed by using LCR meter 4080 (Voltcraft LCR 4080, Conrad Electronic, Hirschau, Germany), which was operated in parallel mode at an alternating frequency of 120 Hz (error at kΩ values < 0.5%). The measurements were made by placing the tips of the measuring probes in the donor, and the acceptor chambers were filled with PBS pH 7.4 buffer and separated with a skin sample. Skin samples with impedance > 3 kΩ were used in the research, which corresponds to the electrical resistance of human skin [21].

### 2.8. Statistical Analysis

Each experiment was repeated three times, and the results were presented as the mean value with standard deviation. A one-way analysis of variance (ANOVA) was used. Turkey’s t-test was performed to observe any significant difference between individual groups (α < 0.05). To determine similarities in all compounds tested, a cluster analysis was evaluated. A Pearson test was used to determine the correlation between human skin and Strat-M™ membranes.

## 3. Results and Discussion

The penetration of drugs, topically applied, is restricted by SC, consisting mainly of lipids [5]. Therefore, in the penetration study, the selection of an appropriate substrate may be the key to increasing penetration and achieving a fast therapeutic effect in the tissues under the skin. In addition, the penetration of active substances is influenced by factors such as the physical properties of the drug, including its lipophilicity, solubility, and molar mass and the characteristics of a formulation/vehicle or a drug delivery system. [3]. Another method to increase penetration is also the modification of the active substance, consisting primarily in increasing its lipophilicity [2,6]. In present in vitro study, the penetration of new ibuprofen derivatives was compared with the penetration of pure ibuprofen. The donor phase was a gel with an active substance content of 5% (expressed as content of ibuprofen), while the acceptor phase was a buffer solution of pH 7.4. In our study, the donor phase was Celugel^®^, which was selected for its composition enhancing the penetration of active substances [5]. It is well known that the change in the lipophilicity of active substances affects the permeability and, thus, the speed of action associated with the achievement of the therapeutic concentration of the drug [11]. L-valine alkyl ester ibuprofenates are characterized by higher solubility in water and phosphate buffer (both pH 5.4 and 7.4) than the unmodified ibuprofen and by lipophilicity depending on the length of the alkyl ester chain [2,6].

In our previous work, it was shown that the obtained structural modification of ibuprofen significantly improves the permeability of ibuprofen through the skin from alcoholic solutions. In the presented research, we presented the results of the penetration of IBU and its derivatives from a ready-made commercial carrier in the form of a hydrogel. Hydrogels are a physicochemical system that makes the most of water and gelling polymers [19]. They are used in many fields, particularly in medicine and pharmacy. Hydrogels are characterized often by good and favorable physicochemical properties and biocompatibility. In addition, one of the main advantages of hydrogels used in topical treatment is their ease of application and significant minimization of side effects [18].

**Figure 2:**
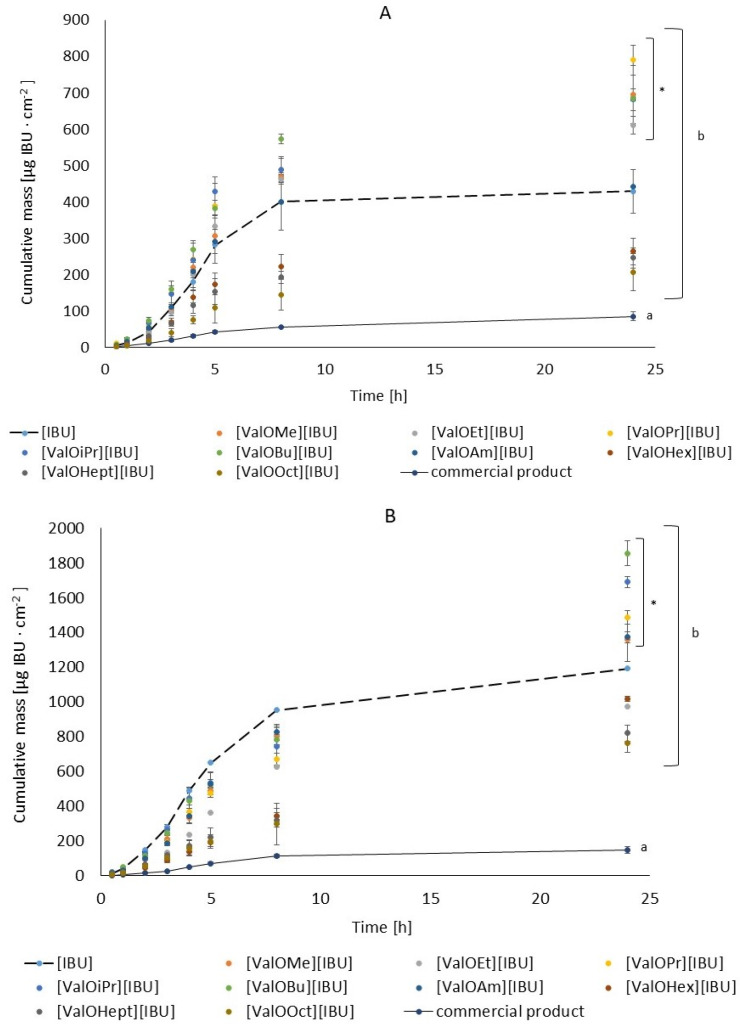
Time course of the cumulative amount of [IBU] and its modifications that permeated through human skin (**A**) and Strat-M™ (**B**). Five percent IBU and its derivatives in Celugel^®^ and commercial product single application 1 g·cm^−2^ of human skin and Strat-M™. Each point represents the mean ± SD (*n* = 3). For * *p* < 0.0001 versus the control (pure ibuprofen). Different letters also mean essential between Celugel^®^ (b) and commercial product (a).

Table 2 summarizes the amounts of compound (expressed in IBU amount) in the acceptor fluid after 24 h of permeation testing. The cumulative mass in acceptor fluid, considering all time points, is shown in Figure 2A,B. The cumulative mass of the [IBU] in the acceptor phase after 24 h of permeation was significantly higher in the case of Celugel^®^ application containing [ValOiPr][IBU] (790.52 μg IBU·cm^−2^) > [ValOMe][IBU] (696.68 μg IBU·cm^−2^) > [ValOBu][IBU] (684.54 μg IBU·cm^−2^) > [ValOPr][IBU] (682.20 μg IBU·cm^−2^) and [ValOEt][IBU] (611.44 μg IBU·cm^−2^) compared to the application of the control (rac-IBU) (429.67 μg IBU·cm^−2^). On the other hand, in the case of Strat-M™ membranes, they were as follows: [ValOBu][IBU] (1856.66 μg IBU·cm^−2^) > [ValOPr][IBU] (1691.71 μg IBU·cm^−2^) > [ValOiPr][IBU] (1488.85 μg IBU·cm^−2^) > [ValOAm][IBU] (1374.14 μg IBU·cm^−2^) and [ValOEt][IBU] (1359.35 μg IBU·cm^−2^) compared to the application of the control (1194.36 μg IBU·cm^−2^). For comparison purposes, in our study, the penetration of IBU from a commercial product with the same active substance concentration was also performed in the studies. In this case, the penetration was significantly lower (Table 2). Testing IBU penetration from various vehicles, including the hydrogels, was also performed in other studies. For example, Bolla et al., showed that the cumulative amount of IBU permeated at the end of 24 h through Strat-M™ membrane was highest for clear gel in the compared cream. The enhanced permeation could probably be due to the lower viscosity of the gel compared with emulsion. Additionally, a high thermodynamic activity could have resulted in enhanced permeation of IBU. The high thermodynamic activity was caused by dissolving the drug before releasing it into the medium; therefore, according to the authors, this method of preparation is definitely more beneficial [3].

**Figure 3:**
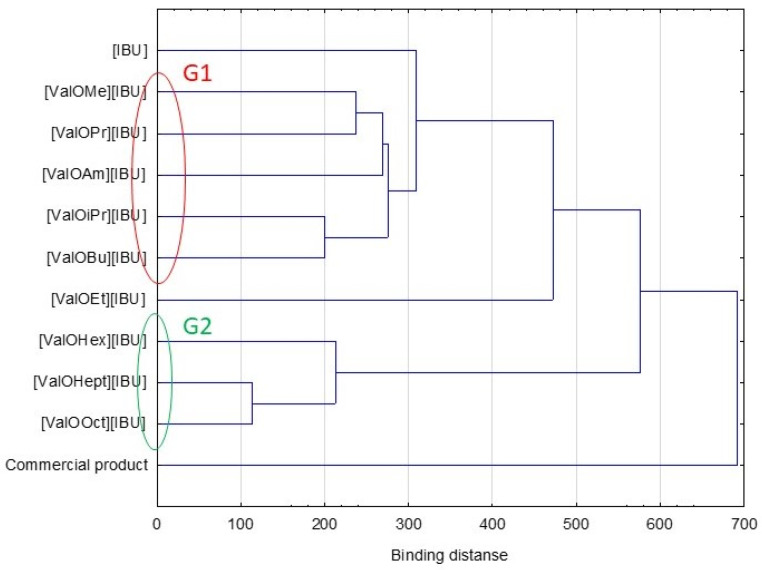
Cluster analysis plot for mean cumulative mass for [IBU] and its derivatives permeated through human skin and Strat-M™ from hydrogel preparations.

**Figure 4:**
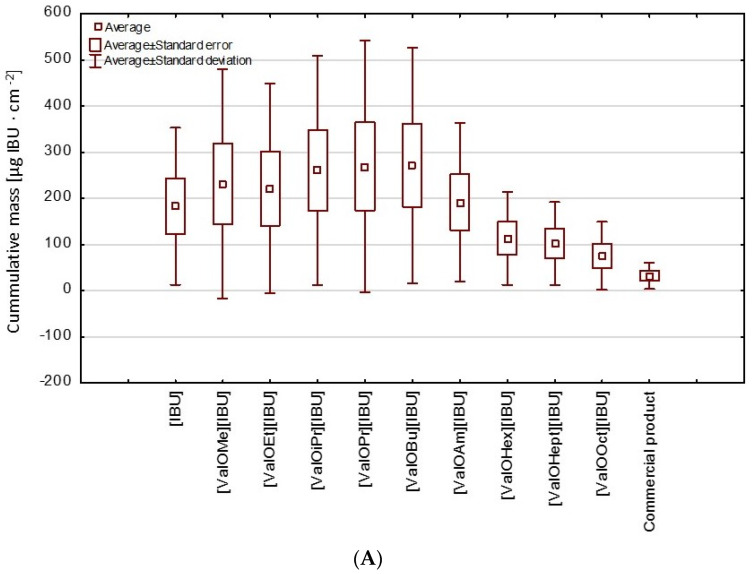

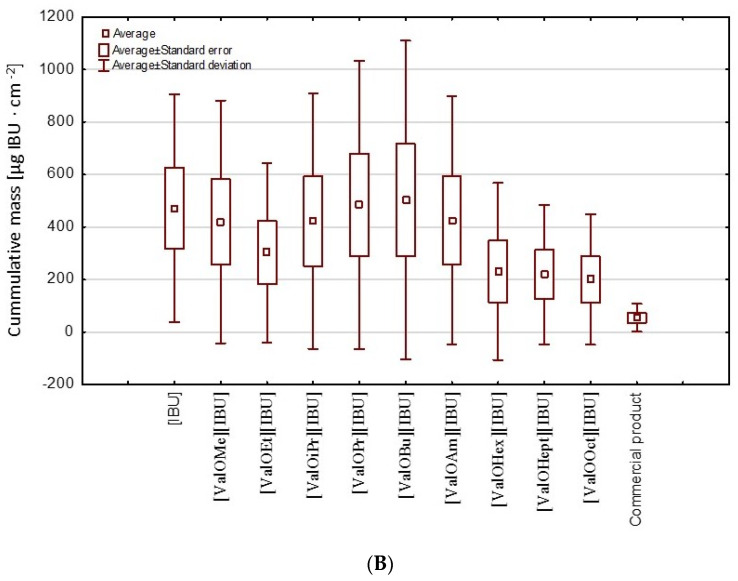
A box plot of the average cumulative mass of [IBU] and its derivatives after 24 h penetration from hydrogel preparations through (**A**) human skin and (**B**) Strat-M™ membranes.

This study has also performed cluster analysis to establish the similarities between individual compounds throughout the 24 h study period. Two clear groups in the cluster analysis graph were observed (Figure 3). The IBU derivatives such as [ValOiPr][IBU], [ValOMe][IBU], [ValOBu][IBU], and [ValOPr][IBU] were characterized by a similar penetration, which was significantly higher in the compared control (rac-IBU). These compounds formed the first group (red circle—G1). While a separate group characterized by lower penetration compared to pure [IBU] consists of [ValOHex][IBU], [ValOHept][IBU], and [ValOOct][IBU] (green circle—G2). The higher penetration of [ValOiPr] [IBU], [ValOMe] [IBU], [ValOBu] [IBU], and [ValOPr] [IBU] is also confirmed by box plots (Figure 4).

The higher penetration of [ValOiPr][IBU], [ValOMe][IBU], [ValOBu][IBU], and [ValOPr][IBU] used in our study is related to their modified chemical properties. Our previous research has shown that the new [IBU] derivatives penetrated in greater amounts through the pigskin than [IBU]. In our previous studies, the effect of various alcohols as vehicles on skin permeability was compared for ion pairs of ibuprofen with L-valine alkyl esters [ValOR][IBU], in which the alkyl chain R was changed from C1 to C8 in compared pure ibuprofen [2,6]. The acceptor solution was the alcohols, such as methanol, ethanol, and isopropanol. Due to the structural modification of ibuprofen in the form of an amino acid alkyl ester, the compound’s solubility in water and its permeability through the skin increased. [6]. Furthermore, the derivatives such as [ValOiPr][IBU], [ValOPr][IBU], and [ValOBu][IBU] [ValOAm][IBU] are characterized by significantly lower hydrophobicity compared to pure IBU. The saturation concentration was in the range between 3.055 g IBU dm^−3^ for [ValOEt][IBU] and 0.259 g IBU dm^−3^ for [ValOOct][IBU] in the buffer of pH 7.4 [6]. The pH 7.4 of the acceptor fluid was also used in this study. The solubility of the amino acid alkyl ester salts was 1.9 times higher for [ValOOct][IBU] to 42.8 times higher for [ValOEt][IBU] than for unmodified [IBU]. In our study, it can be observed to be increasing when the solubility increases the permeability. However, we should remember solubility–permeability tradeoff. The most optimal one is to find solubility–permeability balance. These studies allowed for the selection of modifications that provide the best results.

According to the study of the Wenkers and co., the skin permeability for anti-inflammatory drugs depends on the hydrophilicity of these drugs. The authors investigated the penetration of various anti-inflammatory drugs, including ibuprofen. For their research, they used a lipophilic carrier—light mineral oil. As their research shows, the permeability of the human skin for these drugs contained in a lipophilic vehicle is a function of their hydrophilicity, while the maximum flux is primarily dependent on their vehicle solubilities [22]. The change in the lipophilicity of the compound affects the penetration of active substances into the skin. Due to the lipophilic nature of the stratum corneum, the penetration of active substances is limited, and the increase in lipophilicity of the active compound may accelerate its penetration [17,23].

The fluxes of [IBU] and its modifications through the skin (J_SS_), the diffusion coefficient (K_P_), and the time required to reach steady-state permeation (lag time–L_T_) were estimated. The permeation parameters were obtained from typically J-shaped profiles by using the following equation:

(1) $$A=J_{\mathrm{ss}}(t-L_{T})$$

where A is the cumulative amount (in μg IBU·cm^−2^) of [IBU] and its salts permeating into the receptor compartment, J_ss_ is the steady-state flux (in μg∙cm^–2^∙h^–1^) t is the time (h), and L_T_ is the lag time (h). The steady-state flux was estimated from the slope of the linear portion of the plot of cumulative mass in the acceptor phase over time. The lag time (L_T_) was determined from the x-intercept of the linear portion of the plot of cumulative mass in the acceptor phase over time and was used to calculate the diffusion coefficient (K_P_) as follows:

(2) $$K_{P}=J_{\mathrm{ss}}/C$$

where C is the concentration in donor phase.

The permeation parameters were obtained from the typically J-shaped profiles using Equations (1) and (2) and are listed in Table 3. The lowest ibuprofen flux was obtained for commercial product, and it was correspondingly 10.54 ± 0.67 μg∙cm^−2^∙h^−1^ through human skin, while through Strat-M™ it was 19.39 ± 2.34 μg∙cm^−2^∙h^−1^. Ibuprofen flux for obtained modification ranged from 30.82 ± 7.96 μg∙cm^−2^∙h^−1^ for [ValOOct][IBU] to 125.53 ± 9.69 μg∙cm^−2^∙h^−1^ for [ValOiPr][IBU] through the human skin, while through Strat-M™ they were were ranged from 48.24 ± 6.79 μg∙cm^−2^∙h^−1^ for [ValOOct][IBU] to 172.63 ± 2.69 μg∙cm^−2^∙h^−1^ for unmodified [IBU]. The improvement of the ibuprofen permeation rate was achieved by introducing the following cations: [ValOMe], [ValOEt], [ValOiPr], [ValOPr], [ValOBu], and [ValOAm]. Obtained modifications of ibuprofen showed the faster permeation of [IBU] through the human skin and a higher diffusion coefficient. By analyzing all derivatives applied on human skin, [ValOiPr][IBU] showed significantly higher steady-state flux as well as permeability coefficient and lag time compared to unmodified [IBU]. The modification of [IBU] had no effect on the lag times since they were similar for all of the analyzed compounds. In our previous studies, a similar result was observed from alcohol solution, where the flux for the ValOPr, ValOiPr, ValOBu, or ValOAm derivatives was higher compared to the control.

Other examples of the use of ibuprofen salt in order to increase its permeability are also known in the literature. For example, Serveiya et al., showed that the triethylammonium salt of ibuprofen showed greater permeability through the PDMS membrane [24], while Furukawa et al., achieved higher permeability for L-proline ethyl ester ibuprofenate through pig skin [25].

**Figure 5:**
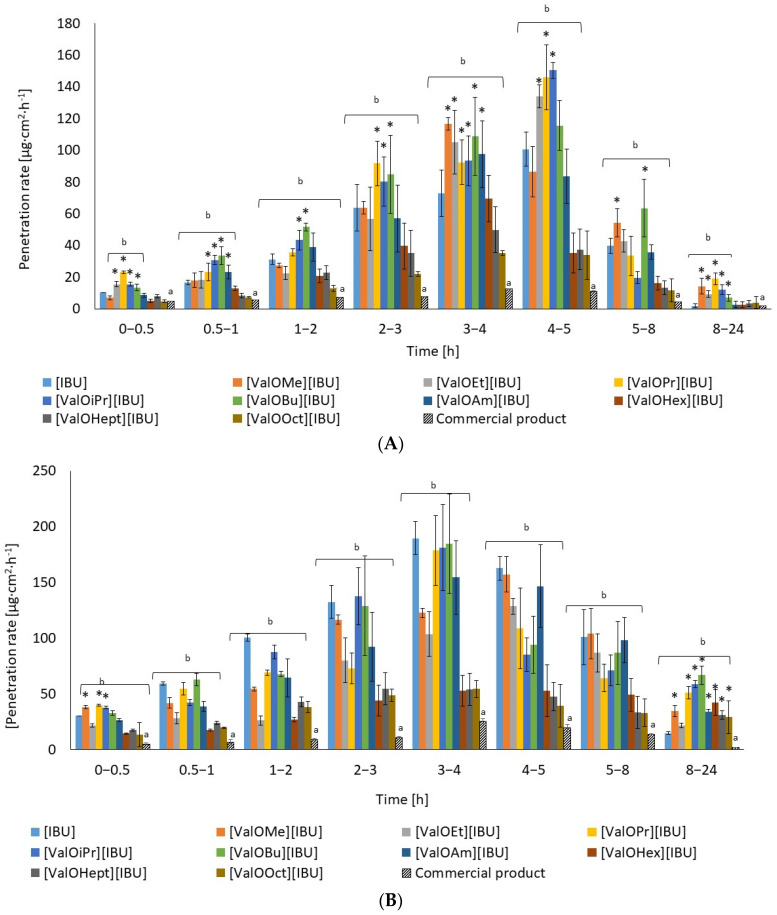
The permeation rate of [IBU] and its modifications and commercial product permeated through human skin (**A**) and Strat-M™ (**B**). All values are presented as mean ± SD where *n* = 3. * *p* < 0.001, in compared unmodified [IBU]. Different letters— significant differences between Celugel^®^ (b) and commercial product (a).

Figure 5 shows the permeation rate of [IBU] and its modifications, which permeated through human skin (A) and Strat-M™ (B). As it can be observed, the highest penetration of tested compounds was generally observed from 3 to 5 h for human skin as well as Strat-M™ membrane.

Synthetic membranes are used for the initial assessment of the permeability of topical drugs due to ethical issues and difficulties in obtaining human skin. Furthermore, human skin is characterized by high biological variabilities such as thickness, hair follicles density, and lipid content [26]. In recent years, there has been a significant rise in using synthetic artificial membranes such as Strat-M™ membranes [3,27]. The Strat-M™ membrane is easy to use, shows lot-to-lot consistency, and does not require any special storage, which in turn simplifies experimental design and data analysis [28]. The outer layer of Strat-M^TM^ consists of two layers of polyethersulfone, while the bottom layer is a more diffusive polyolefin layer [29]. In addition, Strat-M^TM^ contains lipids such as those found in the human *stratum corneum* (SC). Strat-M^TM^ membranes are a very good alternative to natural membranes. The similarity of hydrophobic properties to human skin is due to the presence of synthetic lipids in two of the three polymer layers that make up the membrane, which was confirmed by microscopic analysis [30]. This membrane was used in testing and optimizing pharmaceutical formulations with very good results [27]. Previous works by Uchida et al. [30], Simon et al. [31], Bolla et al. [3], and also Hag et al. [27] confirmed that the Strat-M™ membrane can be successfully used in penetration tests. It has been shown to correlate well with pigskin and even human skin.

**Figure 6:**
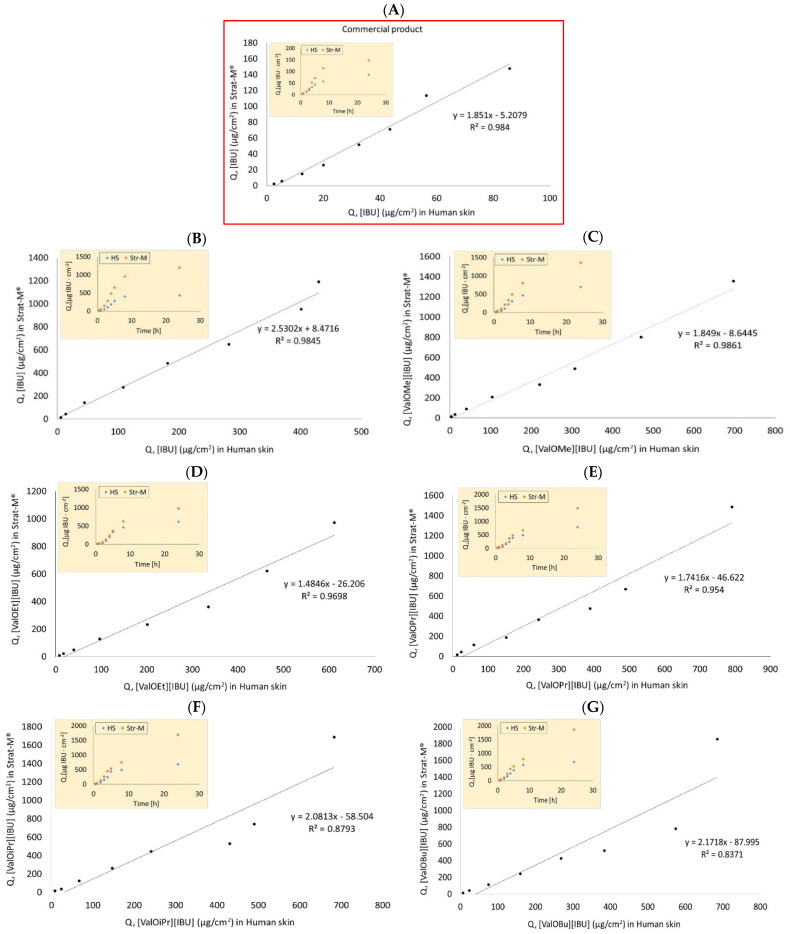

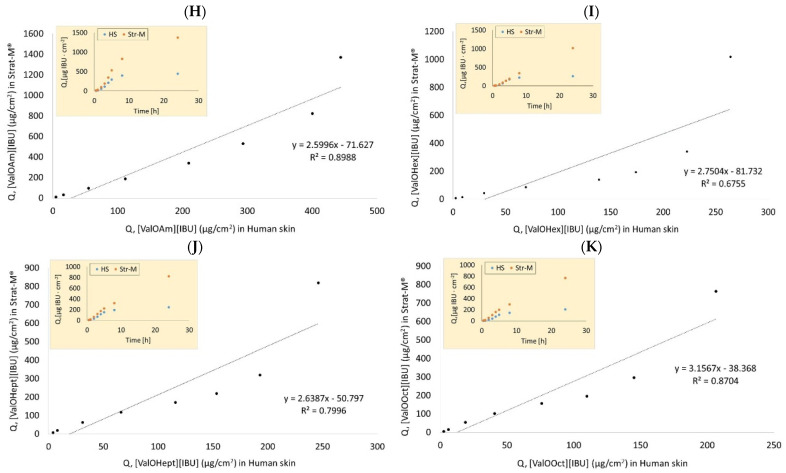
Comparison of mean cumulative mass during 24 h penetration and correlation between human skin and Strat-M™ for all tested formulations: (**A**) commercial product, (**B**) [IBU], (**C**) [ValOMe][IBU], (**D**) [ValOEt][IBU], (**E**) [ValOPr][IBU], (**F**) [ValOiPr][IBU], (**G**) [ValOBu][IBU], (**H**) [ValOAm][IBU], (**I**) [ValOHex][IBU], (**J**) [ValOHept][IBU], (**K**) [ValOOct][IBU]; Pearson’s test, *p* < 0.05.

**Figure 7:**
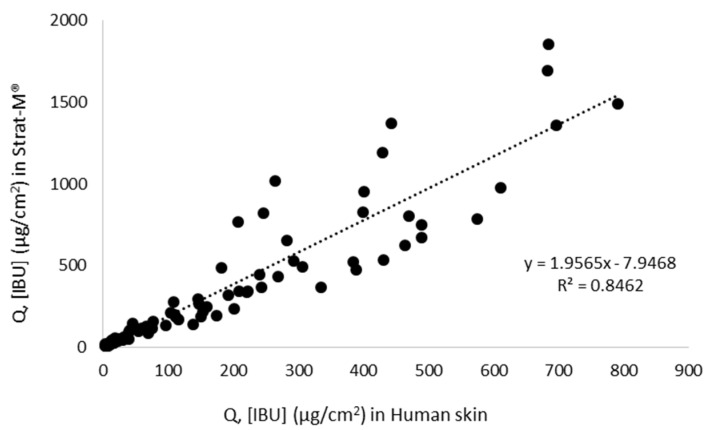
The correlation between human skin and Strat-M™ for all tested compounds during 24 h penetration; Pearson’s test, *p* < 0.05.

In the current study, we compared the penetration of ibuprofen and ibuprofenates of L-valine alkyl esters through human skin and Strat-M™. It was shown that for individual compounds, the correlations ranged from R^2^ = 0.6755 for [ValOHex][IBU] to R^2^ = 0.986 for [ValOMe][IBU]. All these dependencies are presented in Figure 6. Figure 7 shows the relationship between the mean cumulative mass for all analyzed compounds during the 24 h human skin penetration test and Strat-M™. This correlation was also high, R^2^ = 0.8462. Despite the high correlation between Strat-M™ and human skin in most derivatives and free [IBU], it should be noted that the amount of drugs penetrating Strat-M™ in all cases was greater than the amount of drugs in the skin. Similar observations were noted for caffeine [27,32], cortisone, sodium diclofenac, mannitol, salicylic acid, and testosterone [32]. This is most likely related to the fact that the Strat-M™ does not have organized SC intercellular structures. Its structure is not as complex and heterogeneous as natural skin [33].

The diffusion coefficient is influenced not only by the nature of the molecules but also by the membrane used for the permeation. Therefore, one of the best ways to establish the relation of the artificial membrane is to compare the similarity of penetration relative to the skin [29].

**Figure 8:**
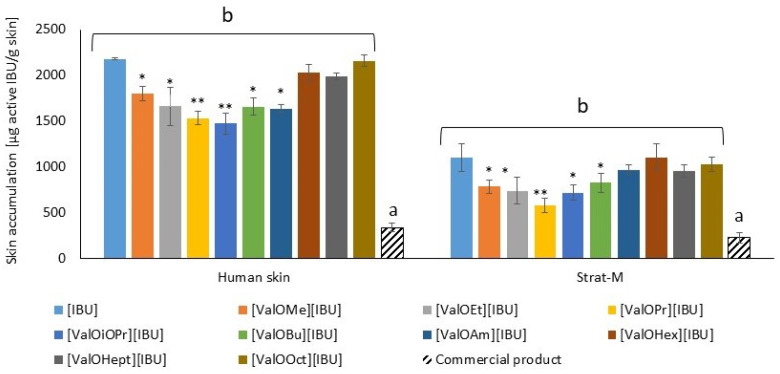
Skin accumulation after 24 h skin penetration of [IBU] and its modification from Celugel formulation and a commercial product. All values are presented as mean ± SD where *n* = 3. * *p* < 0.001, ** *p* < 0.0001 in compared unmodified [IBU]. Different letters—significant differences between Celugel^®^ (b) and commercial product (a).

Additionally, ibuprofen and the ibuprofenates of L-valine alkyl esters accumulation in human skin and Strat-M™ were investigated. Figure 8 shows the amount of accumulated [IBU] in human skin and Strat-M™ membranes after 24 h, expressed in μg IBU g^−1^ of skin. In general, ibuprofen salts showed less tendency to accumulate in the skin compared to unmodified ibuprofen. The longer the alkyl chain of the ester, the higher the accumulation in the skin. A significant difference was shown between the accumulation of IBU from Celugel^®^ and the commercial product (Figure 8). The accumulation in Strat-M™ was relatively lower than in human skin.

## 4. Conclusions

In this study, the influence of changes in the structure of ibuprofen on its permeability from hydrogel preparations was investigated. The obtained results were compared to a commercial preparation containing [IBU] in the tested dose. Our research has shown that Celugel^®^ can be a very good carrier of non-steroidal anti-inflammatory drugs such as ibuprofen and can be successfully used in preparations applied to the skin. Compared to the commercial preparation, hydrogels containing both ibuprofen and its salts showed higher penetration of the active substance. Moreover, it has been shown that the skin permeability of some L-valine ester salts is significantly higher than that of unmodified [IBU]. The best results were obtained with the cations [ValOiPr], [ValOPr], and [ValOBu]. In addition, it is anticipated that modifications of ibuprofen in the form of L-valine alkyl ester salts, in addition to the therapeutic effect specific for ibuprofen, may become an excellent source of this exogenous amino acid. Moreover, the action of ibuprofen may be enriched with the action is attributed to L-valine. L-valine is involved in many processes, including triggering gluconeogenesis or inhibiting the degradation of muscle-building proteins. The use of Celugel^®^ as a transdermal carrier in conjunction with the resulting drug modification may be an excellent proposition for greater penetration and a faster therapeutic effect. Additionally, it has been shown that Strat-M^TM^ membranes can serve as a replacement for human skin in drug permeation and accumulation studies.

## Figures and Tables

**Table 1:** Composition of obtained hydrogels.

Compound	The Molar Mass of Used the Compound (g·mol^−1^)	Pharmaceutical Vehicle (g)	Compound (g)	Ethanol (g)	Total (g)
[IBU]	206.284	0.850	0.0500	0.100	1.00
[ValOMe][IBU]	337.458	0.850	0.0820	0.100	1.03
[ValOEt][IBU]	351.485	0.850	0.0859	0.100	1.04
[ValOiPr][IBU]	365.512	0.850	0.0888	0.100	1.04
[ValOPr][IBU]	365.512	0.850	0.0891	0.100	1.04
[ValOBu][IBU]	379.539	0.850	0.0910	0.100	1.04
[ValOAm][IBU]	393.565	0.850	0.0962	0.100	1.05
[ValOHex][IBU]	405.576	0.850	0.0991	0.100	1.05
[ValOHept][IBU]	421.619	0.850	0.1022	0.100	1.05
[ValOOct][IBU]	435.646	0.850	0.1061	0.100	1.06

**Table 2:** The average cumulative mass of [IBU] and its modification after 24 h permeation test from the Celugel^®^ and commercial product across human SC and Strat-M™.

Compound	Human Skin	Strat-M™
	Cumulative Mass (μg IBU·cm^−2^)	
[IBU]	429.672 ± 60.151 ^b^	1194.362 ± 41.23 ^b^
[ValOMe][IBU]	696.683 ± 79.909 ^b,^*	1359.355 ± 123.895 ^b,^*
[ValOEt][IBU]	611.438 ± 24.918 ^b,^*	974.981 ± 62.779 ^b^
[ValOiPr][IBU]	790.526 ± 41.426 ^b,^*	1488.846 ± 40.435 ^b,^*
[ValOPr][IBU]	682.201 ± 29.910 ^b,^*	1691.708 ± 30.139 ^b,^*
[ValOBu][IBU]	684.538 ± 5.599 ^b,^*	1856.676 ± 71.953 ^b,^*
[ValOAm][IBU]	443.249 ± 49.597 ^b^	1374.142 ± 32.535 ^b,^*
[ValOHex][IBU]	263.958 ± 36.699 ^b^	1019.904 ± 14.149 ^b^
[ValOHept][IBU]	246.074 ± 27.522 ^b^	821.362 ± 46.555 ^b^
[ValOOct][IBU]	206.491 ± 50.088 ^b^	765.666 ± 54.922 ^b^
**Commercial Product**		
[IBU]	85.737 ± 11.868 ^a^	148.081 ± 19.282 ^a^

All values are presented as mean ± SD, where *n* = 3. * Value is higher significantly from control (ibuprofen) (*p* < 0.0001); different letters also mean significant differences between Celugel^®^ (b) and commercial product (a), α = 0.050.

**Table 3:** Permeation parameters of [IBU] and its modification from Celugel^®^ formulation through human skin and Strat-M™.

Compound	Human Skin			Strat-M™			Permeation Ratio (J_Strat-M_^TM^/J_Skin_)	r^2^ (Q_Strat-M_™ vs. Q_Skin_)
	Jss, μg∙cm^−2^∙h^−^^1^	K_P_·10^3^, cm∙h^−1^	L_T,_ h	Jss, μg∙cm^−2^∙h^−1^	K_P_·10^3^, cm∙h^−1^	L_T,_ h		
**Celugel^®^**								
[IBU]	78.46 ± 9.89 ^b^	1.54 ± 0.04 ^b^	1.52 ± 0.14 ^b^	172.63 ± 2.69 ^b^	3.40 ± 0.05 ^b^	0.81 ± 0.01 ^b^	2.20 ^b^	0.985 ^a^
[ValOMe][IBU]	91.72 ± 14.24 *^,b^	1.81 ± 0.28 *^,b^	1.67 ± 0.03 *^,b^	119.54 ± 5.21 ^b^	2.36 ± 0.10 ^b^	0.86 ± 0.02 ^b^	1.30 ^b^	0.986 ^a^
[ValOEt][IBU]	99.16 ± 9.02 *^,b^	1.95 ± 0.18 *^,b^	1.81 ± 0.03 *^,b^	97.48 ± 4.61 ^b^	1.92 ± 0.09 ^b^	0.62 ± 0.02 ^b^	0.98 ^b^	0.970 ^b^
[ValOiPr][IBU]	125.53 ± 9.69 *^,b^	2.50 ± 0.19 *^,b^	1.79 ± 0.06 *^,b^	139.59 ± 4.99 ^b^	2.75 ± 0.10 ^b^	0.96 ± 0.07 *^,b^	1.11 ^b^	0.879 ^b^
[ValOPr][IBU]	108.26 ± 14.43 *^,b^	2.16 ± 0.29 *^,b^	1.56 ± 0.02 ^b^	126.08 ± 6.86 ^b^	2.48 ± 0.14 ^b^	0.82 ± 0.01 ^b^	1.16 ^b^	0.954 ^b^
[ValOBu][IBU]	103.51 ± 13.86 *^,b^	2.06 ± 0.28 *^,b^	1.36 ± 0.04 ^b^	110.74 ± 1.18 ^b^	2.18 ± 0.02^b^	1.78 ± 0.86 ^b^	1.07 ^b^	0.837 ^b^
[ValOAm][IBU]	81.12 ± 2.35 ^b^	1.61 ± 0.05 ^b^	1.44 ± 0.18 ^b^	125.25 ± 3.55 ^b^	2.47 ± 0.07 ^b^	0.82 ± 0.09 ^b^	1.54 ^b^	0.899 ^b^
[ValOHex][IBU]	50.30 ± 5.98 ^b^	1.00 ± 0.12 ^b^	1.45 ± 0.02 ^b^	50.16 ± 3.71 ^b^	0.99 ± 0.07 ^b^	0.84 ± 0.09 ^b^	0.99 ^b^	0.676 ^b^
[ValOHept][IBU]	43.28 ± 6.85 ^b^	0.86 ± 0.14 ^b^	1.30 ± 0.04 ^b^	54.45 ± 11.20 ^b^	1.07 ± 0.22 ^b^	1.40 ± 0.44 *^,b^	1.26 ^b^	0.800 ^b^
[ValOOct][IBU]	30.82 ± 7.96 ^b^	0.61 ± 0.16 ^b^	1.50 ± 0.15 ^b^	48.24 ± 6.79 ^b^	0.95 ± 0.13 ^b^	1.20 ± 0.04 *^,b^	1.56 ^b^	0.870 ^b^
**Commercial Product**								
[IBU]	10.54 ± 0.67 ^a^	0.21 ± 0.01 ^a^	1.14 ± 0.43 ^a^	19.39 ± 2.34 ^a^	0.39 ± 0.05 ^a^	0.737 ± 0.06 ^a^	1.84 ^a^	0.984 ^a^

J_SS_—steady-state flux; K_P_—permeability coefficient; L_T_—Lag time; r^2^—correlation coefficient. The values are presented as mean ± SD, where *n* = 3. * Value is higher significantly from control (ibuprofen) (*p* < 0.001), different letters also mean significant differences between Celugel^®^ (b) and commercial product (a), α = 0.050.

## Data Availability

The data presented in this study are available on request from the corresponding author.
